# *VARS1* mutations associated with neurodevelopmental disorder are located on a short amino acid stretch of the anticodon-binding domain

**DOI:** 10.55730/1300-0152.2631

**Published:** 2022-12-05

**Authors:** Semra HIZ, Seval KILIÇ, Güney BADEMCİ, Tülay KARAKULAK, Aybike ERDOĞAN, Burcu ÖZDEN, Çiğdem ERESEN, Esra ERDAL, Uluç YİŞ, Mustafa TEKİN, Gökhan KARAKÜLAH, Ezgi KARACA, Mehmet ÖZTÜRK

**Affiliations:** 1 İzmir Biomedicine and Genome Center, İzmir, Turkey; 2 Department of Pediatrics, Faculty of Medicine, Dokuz Eylül University, İzmir, Turkey; 3 Dokuz Eylül University, İzmir International Biomedicine and Genome Institute, İzmir, Turkey; 4 John P. Hussman Institute for Human Genomics and Dr. John T. Macdonald Foundation Department of Human Genetics, University of Miami Miller School of Medicine, Miami, USA; 5 Department of Medical Biology and Genetics, Faculty of Medicine, Dokuz Eylül University, İzmir, Turkey

**Keywords:** VARS1, whole exome sequencing, protein stability, structural modeling, mutation modeling, psychomotor retardation

## Abstract

Majority of 37 human aminoacyl tRNA synthetases have been incriminated in diverse, mostly recessive, genetic diseases. In accordance with this, we uncovered a novel homozygous valyl-tRNA synthetase 1 (*VARS1*) gene variant, leading to p.T1068M mutation. As in the previously reported *VARS1* mutations, the affected individual harboring p.T1068M was experiencing a neurodevelopmental disorder with intractable seizures, psychomotor retardation, and microcephaly. To link this phenotypic outcome with the observed genotype, we structurally modeled human *VARS1* and interpreted p.T1068M within the spatial distribution of previously reported *VARS1* variants. As a result, we uncovered that p.T1068M is clustered with three other pathogenic mutations in a 15 amino acid long stretch of the VARS1 anticodon-binding domain. While forming a helix-turn-helix motif within the anticodon-binding domain, this stretch harbors one-fourth of the reported *VARS1* mutations. Here, we propose that these clustered mutations can destabilize the interactions between the anticodon-binding and the tRNA synthetase domains and thus hindering the optimal enzymatic activity of *VARS1*. We expect that the depiction of this mutation cluster will pave the way for the development of drugs, capable of alleviating the functional impact of these mutations.

## 1. Introduction

Aminoacyl tRNA synthetases (ARSs) are ancient housekeeping genes. ARSs are ubiquitously expressed in all cells to catalyze the esterification of amino acids with their specific tRNA. Such specific tRNAs carry an appropriate anticodon sequence, which will be translated to messenger RNAs for the high fidelity production of proteins (Schimmel, 2018). There are 37 different human ARSs encoded by different genes. Majority of these ARSs are located either in the cytoplasm or in the mitochondria (except three functioning in both compartments) (Antonellis and Green, 2008). As reviewed in 2017, 31 ARS genes have been incriminated in different, mostly recessive, genetic diseases (Meyer-Schuman and Antonellis, 2017). Most of these profiled mutations show loss-of-function effects, leading to serious problems during protein translation. There are also examples where the ARS mutations can be tolerated, showing that such disease-causing missense variants may retain some residual activity (Meyer-Schuman and Antonellis, 2017).

Among the ARS genes, two homozygous Valine ARS (*VARS1*) variants were first identified in two individuals affected by neurological disorders (Karaca et al., 2015). Both individuals had severe developmental delay, microcephaly, seizures, and cortical atrophy. Expanding on this study, we screened a small set of Turkish families composed of consanguineous couples with children having neurological disorders. Through whole exome sequencing (WES), we detected a novel homozygous *VARS1* variant i.e. p.T1068M, in an affected individual who was experiencing microcephaly, developmental delay, and drug-resistant seizures. The p.T1068M, *VARS1* variant is located in close vicinity of the pathogenic p.R1058Q (Karaca et al., 2015), as well pathogenic p.M1064I and p.F1072L mutations (Okur et al., 2018; Stephen et al., 2018). All these mutations are all localized in a helix-turn-helix motif of the anticodon-binding domain, suggesting a functionally critical region for this enzyme. To interpret the effect of these mutations on the function of *VARS1*, we structurally modeled human p.R1058Q; p.M1064I; p.T1068M; p.F1072L *VARS1* bound to their tRNA by using *Thermus thermophilus VARS* as a template. As a result, we observed that these clustered mutations could lead to the destabilization of the interactions between anticodon-binding and catalytic tRNA synthetase domains, thus to a potential loss-of-function.

## 2. Methods

### 2.1. Ethics statement

The ethics committee approval for this study was granted by the Dokuz Eylül University Ethics Committee (document id: 446-SBKAEK). We also took a written consent from the parents of the affected individual.

### 2.2. Next generation whole exome sequencing

The genomic DNA isolation from blood was performed with Invitrogen PureLink Genomic DNA kit (Cat #: K1820-01) according to the manufacturer’s protocol. Isolated DNA of the proband, one sister, mother, and father were randomly chopped into smaller fragments by sonication, then electrophoresed on 1% agarose gel. Fragments with 400–500 bp length were selected. The fragments corresponding to protein-coding exons were purified with Agilent SureSelect kit. Libraries for Illumina platform were prepared, the optimized barcode sequences were added. By this, four to five libraries could be sequenced in one sequencing lane. The sequencing was carried out in Illumina HiSeq2500 platform at TÜBİTAK Marmara Research Center DNA Services Facility (Gebze, Turkey) where the relevant FASTQ files were obtained. Sequencing reads were aligned to GRCh37/hg19 human reference genome with BWA (v.0.7.16a) (Li and Durbin, 2010). The variant calling step was performed with HaplotypeCaller of Genome Analysis Tool Kit (GATK; v.3.6.0) (DePristo et al., 2011). SnpEff package (v.4.1) was used for variant annotation (Cingolani et al., 2012). The variants with low depth of coverage (<7) and quality score (<30) were not considered for the downstream analysis. For the filtering, the variants that were homozygous in the proband and heterozygous in each parent were considered. Homozygous variants in the unaffected sibling were removed. The minor allele frequency (MAF) was chosen to be <0.005 in the global allele frequency of the Genome Aggregation Database (gnomAD; http://gnomad.broadinstitute.org/). The remaining variants were then prioritized according to their mammalian conservation (GERP and PhyloP), predicted deleteriousness (SIFT, PolyPhen2 and MutationTaster) and putative impact (high, moderate, or low).

### 2.3. Sanger validation and segregation analysis by restriction enzyme analysis

The genomic location of candidate variant detected by the next generation WES was amplified by polymerase chain reaction (PCR) via appropriate primers for Sanger validation and restriction digestion. For confirming the WES results, primers covering the mutation site were designed to amplify 488 bp DNA fragment by PCR using high fidelity Phusion polymerase. The amplified fragment of proband was sent to Sanger Sequencing (TRiOGEN) for forward and reverse reading. The sequences were investigated with FinchTV 1.4.0 program. The variant site is a recognition site for the *Tsp*45I digestion enzyme (New England Biolabs ref no: R0583S), which stalled the digestion of the proband DNA. Therefore, the parental and other two sisters’ DNA restriction digestion was used for confirmation. For the restriction reaction, the conditions were set with 10× CutSmart buffer (New England Biolabs #B7204S), in 20 mL, which was incubated at 65 °C 1 h according to manufacturer’s directions. One percent agarose gel electrophoresis was performed to detect the segregated DNA bands.

### 2.4. Structural modeling of p.R1058Q, p.M1064I, p.T1068M, and p.F1072L *VARS1*

The best structural template to model human *VARS1* was the *Thermus thermophilus* valyl-tRNA Synthetase (pdb id: 1IVS (Fukai et al., 2003), with 52.9% of sequence similarity. By using 1IVS as the template, *VARS1* structural model was constructed with MODELLER (Webb and Sali, 2014). As 1IVS did not contain any structural information on 879–916 amino acid range, this region was excluded during model building. Here, we used standard homology modeling, as AlphaFold2 could not produce a model compatible with RNA binding (Supplementary Figure 1A).

MODELLER was run through the MPI Bioinformatics Toolkit web service (https://toolkit.tuebingen.mpg.de/#/) (Alva et al., 2016). Modeled human *VARS1* was aligned on top of 1IVS with the “align” command of PyMOL (Schrödinger LLC, 2015). The *Thermus thermophilus* tRNA-Val was isolated together with human *VARS1* model. The human VARS1-tRNA complex was refined with HADDOCK 2.2 (Van Zundert et al., 2016). p.R1058Q, p.M1064I, p.T1068M, p.F1072L *VARS1* mutations were imposed one at a time by using the HADDOCK as described at https://www.bonvinlab.org/software/haddock2.4/faq/#what-about-point-mutations.

### 2.5. Measuring the impact of the mutation cluster on *VARS1* stability

The structural impact of the clustered mutations (p.R1058Q, p.M1064I, p.T1068M, p.F1072L) was assessed based on their interdomain contact changes (between the anticodon-binding and tRNA synthetase domains). The contacts were calculated with the PRODIGY web server (Xue et al., 2016). The specific interactions were calculated with Protein Interactions Calculator web server (Tina et al., 2007). The impact of novel p.T1068M and previously reported p.R1058Q, p.M1064I, p.F1072L mutations on the anticodon-binding domain’s stability were calculated with the MAESTROWeb (Laimer et al., 2015). We also used mutfunc web-server to check whether the mutations would lead to loss-of-function (http://www.mutfunc.com).

## 3. Results

### 3.1. Affected individual

A 45-day-old infant presented with migrating myoclonic seizures that had been lasting for three days. The seizures were apparent both during sleep and activity. She had focal tonic and clonic seizures. The infant was delivered to a 39-year-old mother with gestational diabetes without any complication at the 38th week of gestation via caesarean section. Upon her birth, she was weighing 3300 g. She was the third child of healthy parents who were first cousins. Before having her, the parents had two healthy daughters.

The affected individual’s past medical history included two hospitalizations. She was hospitalized the first time when she was three days old due to dehydration and hypernatremia. She was then hospitalized when she was 15 days old due to urinary tract infection with indications of microcephaly, left renal agenesis, and patent foramen ovale. Her physical examination demonstrated that she had microcephaly, bifrontal narrowing, and high palate. She had mild axial hypotonia with slightly increased deep tendon reflexes, bilateral clonus without Babinski sign. She also had mild upper limb spasticity. Her ocular and hearing examinations were normal. Her laboratory examinations were normal with regard to complete blood count, and biochemistry tests (i.e. blood glucose, creatine kinase, lactate, ammonia, hepatic and renal function tests, lipid profile, vitamin B12, thyroid hormones, metabolic screening including urine organic acids, very long chain fatty acids, acylcarnitines, plasma and urine amino acids, lysosomal enzymes, serum lactate and pyruvate, biotinidase activity, cerebrospinal fluid (CSF) amino acid analysis, CSF/serum glycine and glucose ratios, urine sulfite screening). The electroencephalography showed multifocal epileptic activity. The brain magnetic resonance (MR) imaging revealed diffuse T2 hyperintensity in cerebral white matter. The MR spectroscopy indicated minimally increased lactate peak. Skeletal X-ray was normal. Her follow-up examinations at three months of age revealed that her clinical presentation progressed leading to axial hypotonia with increased deep tendon reflexes, clonus, and spasticity. She had severe global developmental delay without head control, eye contact, or tracking too. She had epileptic encephalopathy with drug (clobazam, clonazepam, levetiracetam, phenobarbital, potassium bromide, pyridoxine, topiramate, and ketogenic diet) resistant seizures. She died at the age of 11 months due to complications caused by an upper respiratory infection.

### 3.2. The whole exome sequencing identifies a homozygous missense *VARS1* variant

The WES analysis of the affected individual’s proband revealed eight homozygous variants (Supplementary Table 1). Among these, only the variants observed in *VARS1*, *LAMC2*, and *USP24* genes occurred at conserved positions (for *VARS1*, please see Supplementary Figure 2A). The fact that the previously reported *VARS1* mutations were associated with microcephaly and epilepsy made us focus on the *VARS1* variant. The *VARS1* gene variant is located at the 31747470th nucleotide of chromosome 6 (6:31747470 (GRCh37), NC_000006.11:g.31747470G>A), leading to a p.Thr1068Met (p.T1068M, rsID: rs777665186) substitution (Figure 1A). This variant has previously been observed with 0.00002 worldwide frequency, but it has never been linked to a clinical condition. It was determined to be homozygous in the proband and heterozygous in other family members (see Section 2). Sanger sequencing confirmed proband’s homozygous c.3203G>A variation (Figure 1A). To further validate the genotype of the family concerning the identified mutation, *VARS1* gene around the mutation site was PCR amplified and subjected to *Tsp*45I digestion. The c.3203G>A site prevents the proband’s DNA cleavage by *Tsp*45I, as it coincides with the restriction site of the enzyme (Figure 1B). On the other hand, the parents’ and one unaffected sister’s DNA indicated a pattern compatible with heterozygous mutation, while the other sister contained two healthy alleles (Figures 1C and 1D). Considering the clinical outcome of the previously reported *VARS1* variants (MIM: 617802, phenotype: neurodevelopmental disorder with microcephaly, seizures, and cortical atrophy), together with the condition of our affected individual suggested further exploration of the autosomal recessive p.T1068M *VARS1* mutation.

### 3.3. Anticodon-binding domain of *VARS1* is a mutational hotspot

**Figure 2:**
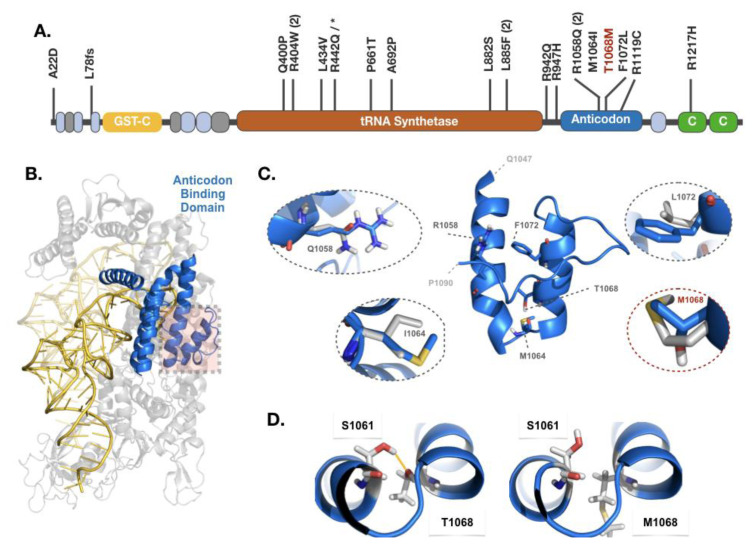
Structural mapping of the pathogenic *VARS1* mutation cluster. **A:** Domain organization of *VARS1* (yellow: glutathione S-transferase, orange: tRNA synthetase, blue: anticodon-binding, green: coiled coil) together with the localization of the reported pathogenic mutations. Light blue and gray boxes correspond to the low complexity regions, as annotated by Pfam (El-Gebali et al., 2019). The novel p.T1068M mutation is depicted in salmon. **B:** The structural model of *VARS1* anticodon-binding domain harboring pathogenic mutations. The color coding follows the one given in panel A. **C:** The structural motif belonging to the wild type anticodon-binding domain (running between amino acids Q1047 and P1090) is located in the middle. The wild type motif is surrounded by the close-up of each observed mutation position. In each oval, the conformation of the mutant amino acid (gray) is compared to its wild type counterpart. **D:** In the wild type *VARS1*, S1061 is making a stabilizing interhelical polar contact with T1068 (depicted by Pymol (Schrödinger LLC, 2015)). This interaction is lost in the case of p.T1068M substitution.

*VARS1* is composed of glutathione S-transferase C-terminal, tRNA synthetase class 1, anticodon-binding and coiled coil domains (Supplementary Figure 1B). So far, including the one we report here, 19 *VARS1* mutations have been described (Figure 2A). Interestingly, 17 of these mutations occurred as homozygous or heterozygous missense mutations, mostly localized on the tRNA synthetase and anticodon-binding domains. A 15 amino acid long stretch of the anticodon-binding domain (encoded by the exon 27 of 207 bp length) harbors our described p.T1068M mutation, as well as the previously reported pathogenic p.R1058Q (recurrent), p.M1064I, and p.F1072L mutations. To observe the spatial distribution of these mutations, we modeled human *VARS1* in complex with its tRNA, by using the valyl-tRNA Synthetase (ValRS) from *Thermus thermophilus* as a template (see Section 2; Supplementary Figure 1B). As a result, we found out that p.R1058Q, p.M1064I, p.T1068M, p.F1072L mutations are clustered within a helix-turn-helix (HTH) motif, running between Q1047-L1075 (Figures 2B and 2C). This HTH motif is located across the anticodon-binding and tRNA synthetase domain interface (Supplementary Figure 1B, Figure 2B). We also explicitly modeled p.R1058Q, p.M1064I, p.T1068M, p.F1072L *VARS1* point mutations (see Section 2). The structural analysis of the mutant *VARS1* structures revealed that each of these four mutations has the potential to reduce the number of (anticodon-binding and tRNA synthetase) domain-domain contacts by 10% (see Section 2; Supplementary Table 2). This reduction is triggered by the loss of interhelical hydrophobic (for p.M1064I and p.F1072M) and electrostatics interactions (for p.R1058Q and p.T1068M). Specifically, in the case of p.T1068M, residue 1068 is unable to make a stabilizing hydrogen bond with p.S1061 located on a reciprocal helix (Figure 2D). These observations were further supported by the MAESTROWeb and mutfunc web-servers, which predicted all of these mutations to be destabilizing/ deleterious (Supplementary Table 3, Supplementary Figure 2B).

## 4. Discussion

Karaca et al. (2015) was the first group reporting two homozygous, *VARS1* gene variants (p.R1058Q and p.L885F, respectively) in two families of consanguineous couples. Three children coming from these families experienced microcephaly, intellectual disability, seizures, and cortical atrophy. Alsemari et al. (2017a, 2017b) then described a homozygous p.R1217H *VARS1* mutation in a boy with severe mental retardation, ataxia, speech impairment, epilepsy, short stature, microcephaly, hypogonadism, and growth hormone deficiency. Stephen et al. (2018) also reported two siblings with severe earlyonset neurological manifestations including microcephaly, intellectual disability, seizures, and cortical atrophy. The affected individuals demonstrated some dysmorphic facial features. WES analysis of these affected individuals detected two mutations in *VARS1*: a missense p.M1064I mutation in one allele and a splice site mutation resulting in a truncation in the other allele. Okur et al. (2018) reported two male siblings aged 15- and 10-year-old who had an intellectual deficiency, developmental delay, severe speech impairment, microcephaly, and prematurity. Both affected individuals were compound heterozygous with p.A22D and p.F1072L mutation in *VARS1* gene. Recently, two further studies were published reporting new *VARS1* variants. Siekierska et al. (2019) described ten affected individuals from seven families with biallelic variants in *VARS1*. All affected individuals demonstrated global developmental delay, microcephaly, and intellectual disability. Eight out of ten affected individuals had epileptic seizures and cerebral atrophy. Friedman et al. (2019) reported seven affected individuals from five unrelated families with five different biallelic missense *VARS1* variants. Subjects presented with global developmental delay, epileptic encephalopathy, primary or progressive microcephaly, and cortical atrophy. While the mutations reported by Siekierska et al. (2019), Friedman et al. (2019), and Stephen et al. (2018) are heterozygous together with a missense mutation, all other known mutations of *VARS1* are biallelic missense mutations (Figure 2A). The lack of affected individuals with a total loss of intact protein strongly suggests that *VARS1* is a vital protein. Thus, missense variants of *VARS1* are probably sufficient to maintain the vital functions, except for the normal development and/or function of the brain tissue. Our affected individual presenting the homozygous p.T1068M *VARS1* mutation has some common features with the abovedescribed affected individuals, including microcephaly, developmental delay, and intractable seizures. Though, she did not have cortical atrophy. In the reports of Okur et al. (2018) and Siekierska et al. (2019), some of the affected individuals did not demonstrate cortical atrophy too. This indicates that cortical atrophy is either not a persistent outcome of *VARS1* mutations or is acquired with time.

When compared with the affected individuals presented in the literature, it can be claimed that the neurological signs and symptoms of our case were more severe. She had axial hypotonia without head control and pyramidal symptoms including increased deep tendon reflexes with bilateral clonus and upper limb spasticity. She had no eye contact, and her microcephaly was prominent. She also had epileptic encephalopathy with refractory myoclonic seizures. Despite the use of many antiseizure drugs and ketogenic diet, her seizures continued. Her death due to an intervening respiratory infection was probably due to problems associated with severe neuromotor retardation and refractory epileptic encephalopathy. Affected individuals living up to 10 years of age have been reported in the literature, but our case died before reaching the age of one. Therefore, we cannot comment on whether symptoms such as cortical atrophy described in older children in the literature will also develop in our patient. One distinctive clinical feature of our affected individual was her left renal agenesis. This finding was not described in any of the affected individual with VARS1 mutation reported in the literature. Since parents of the case were consanguineous as first cousins, the finding of left renal agenesis may be associated with a genetic defect other than VARS1. However, we think that it is important to consider this finding in our case and to report it to the literature. The interpretation of whether this finding is related to VARS1 mutation or an incidental finding can be made when more VARS1 related affected individuals are reported in the literature.

To further explore the impact of the detected mutation, we structurally modeled human *VARS1* and interpreted p.T1068M within the spatial distribution of previously reported VARS1 variants. As a result, we uncovered that p.T1068M is clustered with three other pathogenic mutations (p.R1058Q, p.M1064I, p.T1068M, and p.F1072L) in a 15 amino acid long stretch of the anticodon-binding domain. Further, we showed that all these four mutations lead to the reduction in the number of anticodon-binding and catalytic tRNA synthetase domain-domain interactions by using computational approaches. We supply our models together with this paper as supplementary material to aid the drug design approaches targeting these *VARS1* mutations.

## 5. Conclusion

In this work, we screened a small set of consanguineous Turkish families with children suffering from different diseases, mostly neurological disorders by using whole exome sequencing. This study allowed us to identify a novel *VARS1* variant leading to a p.T1068M mutation. This mutation is located very close to previously reported pathogenic p.R1058Q. Here, we show that a helix-turn-helix motif on the anticodon-binding domain of *VARS1* not only harbors p.T1068M, but also three other pathogenic *VARS1* mutations. We demonstrate that all these mutations are potentially capable of destabilizing anticodon-binding and catalytic tRNA synthetase domain-domain interactions. It is highly likely that such destabilization hinders the optimal enzymatic activity of *VARS1* without compromising vital function. We expect that this realization will pave the way for the development of drugs, capable of enhancing mutant *VARS1* stability.

## Supporting Figures

Supplementary Figure 1 (A) AlphaFold2 model (white) is aligned with *Thermus thermophilus* valyl-tRNA Synt hetase (pdb id: 1IVS, yellow) as the best structural template to homology model *VARS1*. **(B)** The structural model of the human *VARS1* (all depicted in cartoon). The domain coloring follows the scheme reported in Figure 2. tRNA is colored in gray.

Supplementary Figure 2 A. T1068 is conserved across different organisms. **B**. Mutfunc webserver (http://www.mutfunc.com) predicts the amino acids substitutions occurring in the mutation cluster to be impactful due to the conserved positions of these substitutions.

## Supporting Tables

**Supplementary Table 1:** The list of homozygous variants identified in the proband.

Chromosome	Chr Start	Chr End	Var. Type	Ref Seq	Var Seq	rsid List	Genes List	Gene Component List	Prot Impact List	aa Change	Global Allele Freq.	Mammalian Conservation
1	53108597	53108597	SNP (X2)	C	G	rs200885042	FAM159A	CDS	MISSENSE	A-82-G	0.01	no
1	183189995	183189995	SNP (x2)	A	C	LAMC2	CDS	MISSENSE	N-180-T	0	Yes (5.4)	
4	68384019	68384019	SNP (x2)	C	A	CENPC	CDS	MISSENSE	D-229-y	0	no	
6	31747470	31747470	SNP (x2)	G	A	rs777665186	VARS	CDS	MISSENSE	T-1068-M	0.01	Yes (5.3)
12	95927026	95927026	SNP (x2)	T	A	USP44	CDS	MISSENSE	E-336-V	0	Yes (4.9)	
12	101777396	101777396	SNP (x2)	A	G	rs139767850	UTP20	CDS	MISSENSE	M-2669-V	0.02	no
12	104054522	104054522	SNP (x2)	A	T	STAB2	CDS	MISSENSE	Q-617-L	0	no	
12	108961059	108961059	SNP (x2)	T	G	ISCU	INTBQN; CDS	MISSENSE	S-14 5-A	0	no	

**Supplementary Table 2:** Number of contacts calculated by PRODIGY between the anticodon-binding and tRNA synthetase domains, in the case of mutant and wild-type VARS1 (Xue et al., 2016).

Contact types	WT	p.R1058Q	p.M1064I	p.T1068M	p.F1072L
**charged-charged:**	4	3	4	3	3
**charged-polar:**	13	12	12	12	12
**charged-apolar:**	48	46	47	47	44
**polar-polar:**	2	2	2	2	2
**polar-apolar:**	28	29	23	29	24
**apolar-apolar:**	75	73	70	74	78
**Total**	170	165	158	167	163

**Supplementary Table 3:** Stability Predictions calculated by the MaestroWeb web-server for the anticodon-binding domain mutations (Laimer et al., 2015).

Mutation	MaestroWeb (arbitrary units)
**p**.T1068M	0.49 (Destabilizing)
**p**.R1058Q	1.83 (Destabilizing)
**p**.M1064I	0.84 (Destabilizing)
**p**.F1072L	1.76 (Destabilizing)

## Figures and Tables

**Figure 1:**
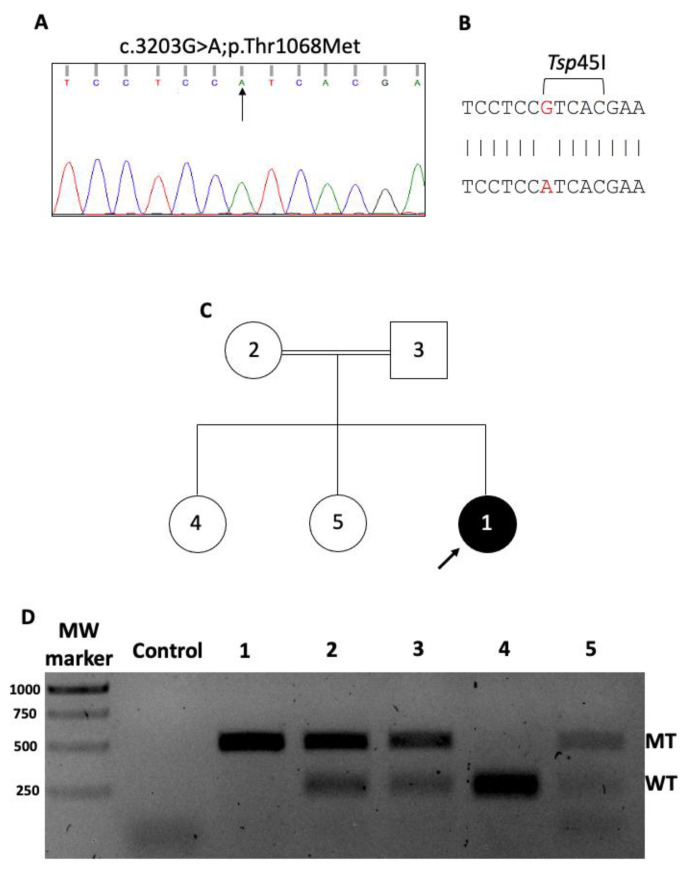
Sanger validation and segregation analysis of *VARS1* variant identified in. **A:** Sanger sequencing of the blood DNA from the proband confirmed homozygous c.3203G>A mutation identified by WES. **B:** The c.3203G>A mutation occurred in a recognition and cutting site by *Tsp*45I restriction enzyme. **C:** The proband (black circled), her parents (2 and 3) and her two unaffected siblings (4 and 5) studied during the segregation analysis by using *Tsp*45I digestion. **D:** Agarose electrophoresis demonstrating homozygous mutation in the proband (MT 488bp), heterozygous mutations in parents and one sister (WT 257/231, MT 488 bp), and wild-type status (WT 257/231) in the other sister.

**Figure 1D f3-turkjbiol-46-6-458:**
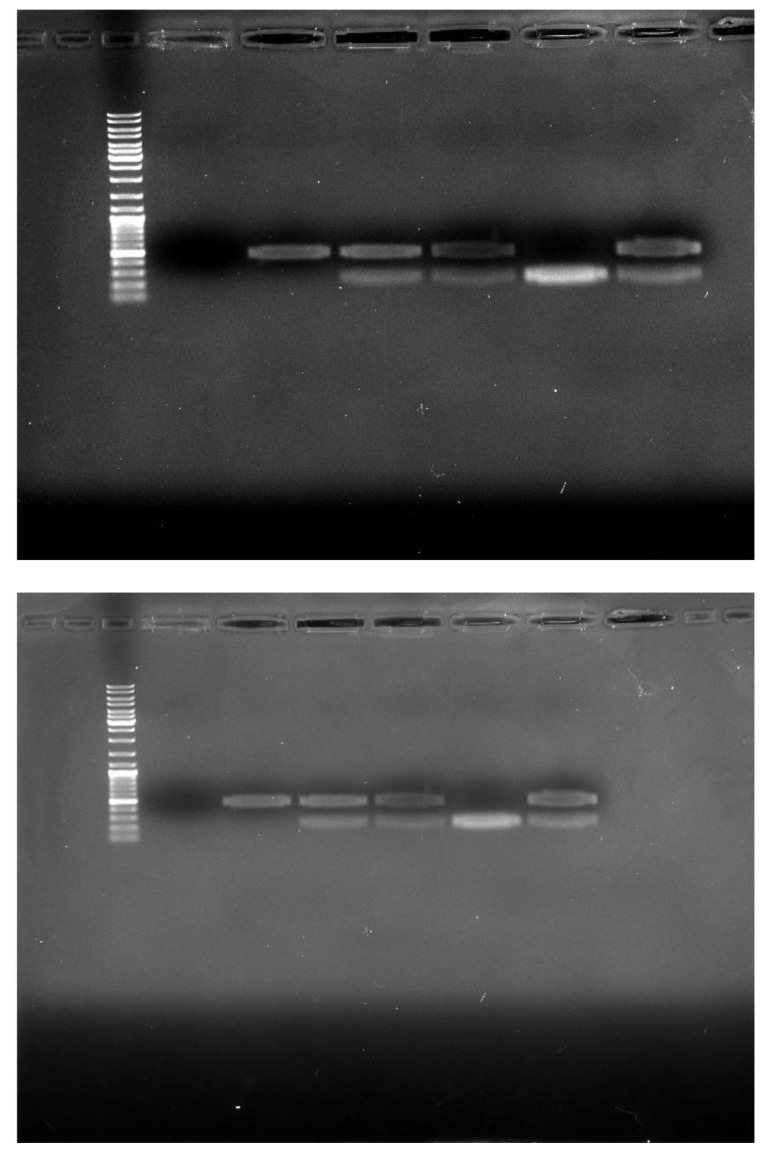
original gel images:

## Data Availability

The atomic coordinates of our models and original gel images are provided as Supporting Information.

## Appendix

[Fig f3-turkjbiol-46-6-458]
