# Supplementary figures and images for: VARS1 mutations associated with neurodevelopmental disorder are located on a short amino acid stretch of the anticodon-binding domain
Source: Turk J Biol. 2022 Dec 5;46(6):458–64. doi: 10.55730/1300-0152.2631 (PMC10388123; doi:10.55730/1300-0152.2631)

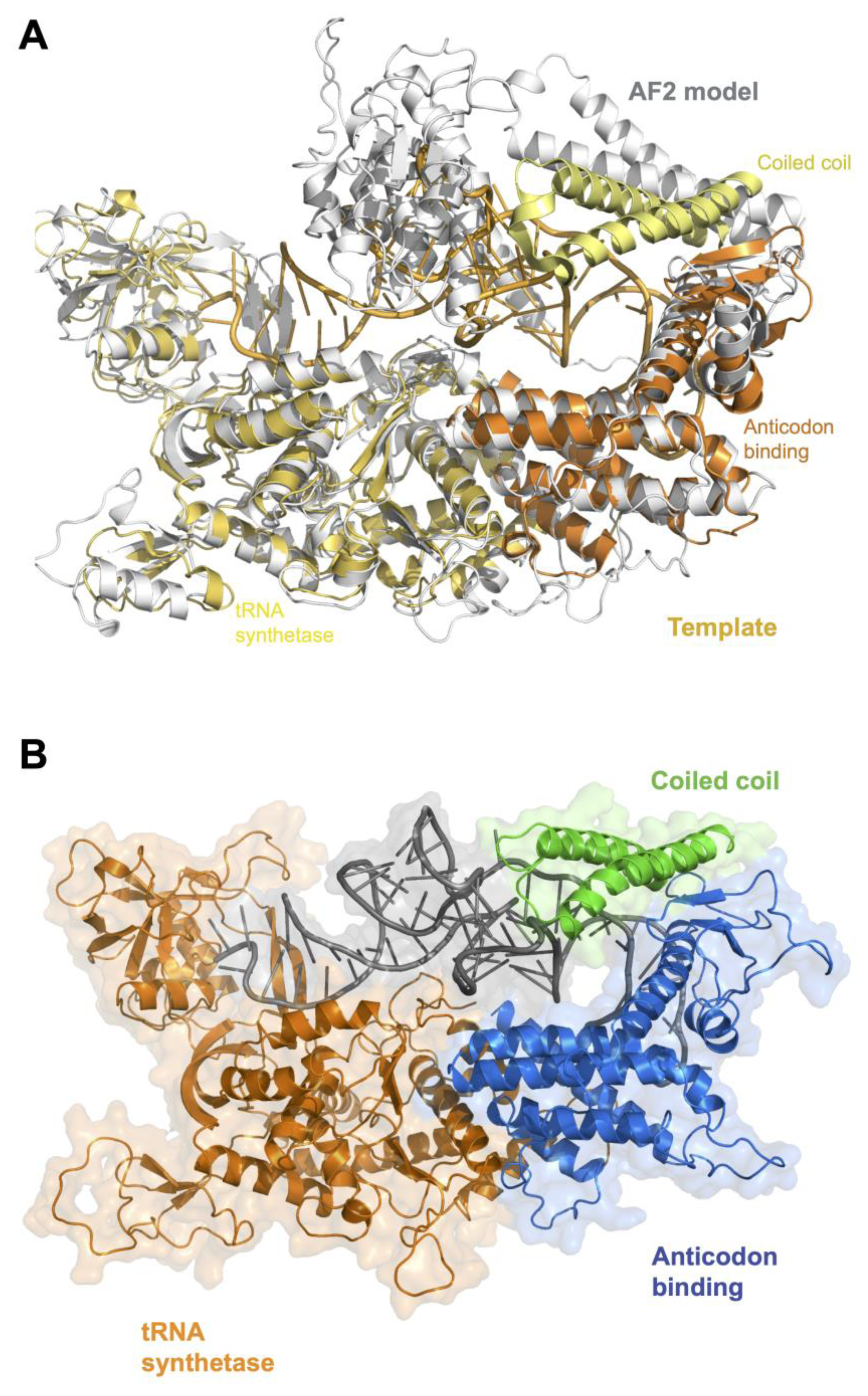

Supplement: Supplementary Figure 1 — (A) AlphaFold2 model (white) is aligned with Thermus thermophilus valyl-tRNA Synt hetase (pdb id: 1IVS, yellow) as the best structural template to homology model VARS1. (B) The structural model of the human VARS1 (all depicted in cartoon). The domain coloring follows the scheme reported in Figure 2. tRNA is colored in gray. [file turkjbiol-46-6-458s1.tif]

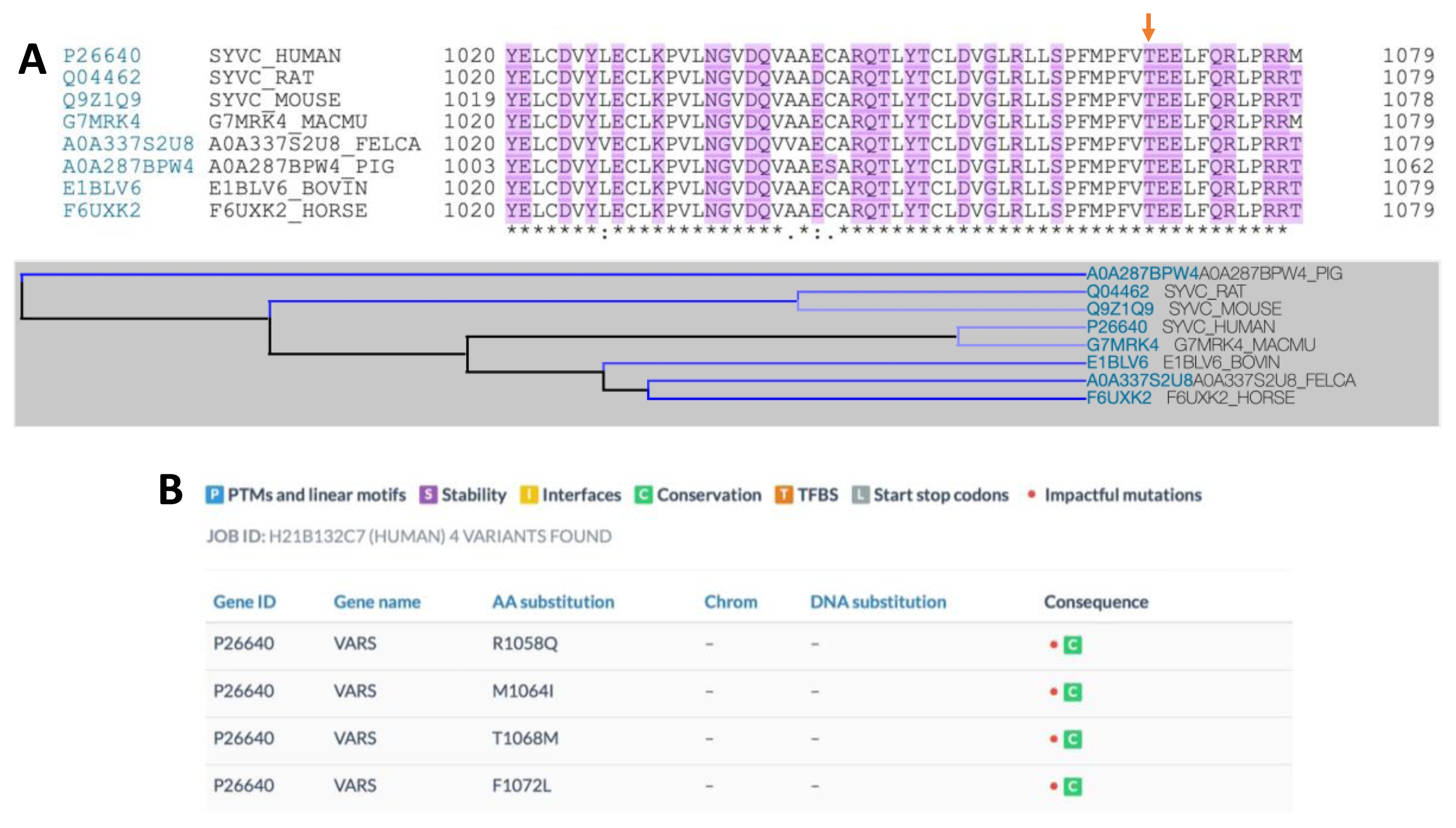

Supplement: Supplementary Figure 2 — A. T1068 is conserved across different organisms. B. Mutfunc webserver (http://www.mutfunc.com) predicts the amino acids substitutions occurring in the mutation cluster to be impactful due to the conserved positions of these substitutions. [file turkjbiol-46-6-458s2.tif]
